# Nasal cavity microbial makeup and the influence on psychiatric symptoms following fire exposure in firefighters

**DOI:** 10.3389/frmbi.2026.1832151

**Published:** 2026-06-23

**Authors:** Paul Grunsted, Chao Xu, Amanda Janitz, Jessica Reese, Janis Campbell, Tasha M. Santiago-Rodriguez, Sara J. Javornik Cregeen, Joseph F. Petrosino, Jooyeon Hwang

**Affiliations:** 1 Department of Biostatistics and Epidemiology, University of Oklahoma Health Sciences Center, Oklahoma City, OK, United States; 2 Baylor College of Medicine, Houston, TX, United States; 3 Department of Environmental and Occupational Health Sciences, University of Texas Health Science Center at Houston, Houston, TX, United States

**Keywords:** firefighter, mental health, microbiome, nasal, psychiatric

## Abstract

**Background:**

Firefighters experience high levels of occupational stress and trauma, increasing their risk of depression, anxiety, and post-traumatic stress disorder (PTSD). Although microbial communities may influence brain function and behavior through neural pathways, the nasal microbiome remains understudied. This study examined associations between nasal microbiome characteristics and psychiatric symptoms among firefighters.

**Methods:**

We conducted a cross-sectional study of 34 firefighters recruited from Texas fire stations. Participants completed validated questionnaires assessing depression, anxiety, and PTSD. Nasal swabs were collected before and after fire suppression and 16S rRNA sequencing was used to characterize microbial communities. Alpha and beta diversity, relative abundance, and differential microbial associations with psychiatric outcomes were assessed using logistic, linear, and linear mixed regression methods.

**Results:**

Sixteen participants (47%) met criteria for depression, six (18%) for anxiety, and four (12%) for PTSD. Alpha diversity was significantly lower in individuals with anxiety (adjusted p = 0.04) while there were no differences in beta diversity or differences in either diversity for PTSD or depression. Increased abundance of the genus *Ruminococcus* was associated with increased odds of anxiety, while *Hydrotalea* was associated with PTSD. Depression scores were positively associated with several genera including *Aerococcus* (1.22; 95%CI: 0.43-2.02) and *Dermabacter* (1.50; 95% CI: 0.37-2.63). Fire suppression was associated with increased *Enhydrobacter* (2.08; 95% CI: 0.80 to 3.46) and decreased *Hymenobacter* (-1.25; 95% CI: -2.22 to -0.27) abundance.

**Conclusions:**

This study identifies preliminary links between nasal microbiome composition and psychiatric symptoms in firefighters and suggests that fire suppression may alter nasal microbial communities.

## 1 Introduction

Firefighters represent a unique and high-risk population for psychiatric disorders, particularly anxiety, depression, and post-traumatic stress disorder (PTSD) (Stanley et al., 2015; Stanley et al., 2016; Martin et al., 2017). Due to the nature of their work, which involves frequent exposure to life-threatening situations, traumatic events, and high-stress environments, firefighters experience significantly higher rates of these psychiatric conditions compared to the general population (Wagner et al., 2021; Obuobi-Donkor et al., 2022). Studies indicate that approximately 30% of firefighters develop mental health conditions during their career, for example, PTSD symptoms occurred in up to 37% of firefighters and depression symptoms can occur in up to 45% of firefighters (Wagner et al., 2021; Russ, 2022; Obuobi-Donkor et al., 2022). Other first responders, such as paramedics and law enforcement officers, also face elevated rates of psychiatric symptoms due to repeated exposure to traumatic events and occupational stress (Russ, 2022; Obuobi-Donkor et al., 2022).

Emerging evidence suggests that chronic stress and traumatic exposures can disrupt various physiological systems, including immune and metabolic pathways, which are closely linked with the body’s microbial communities (Belkaid and Hand, 2014; Xiong et al., 2023; Alotiby, 2024). Anxiety, depression, and PTSD are important public health concerns that impair not only the quality of life of affected individuals but impose substantial socioeconomic burdens, being estimated at over $300 billion, due to healthcare costs, loss of productivity, and associated comorbidities just from depression alone (Konnopka and König, 2020; Arias et al., 2022; Houle et al., 2022; Greenberg et al., 2023). Despite extensive research into these mental health outcomes, the molecular mechanisms underlying these psychiatric conditions remain unclear, hindering the advancement of effective diagnostic and therapeutic strategies. A key challenge in psychiatric research is the lack of insight into the potential biological pathways that contribute to these conditions, since psychiatric conditions are highly complex and multifactorial, involving intricate interactions between genomic, environmental, and biological factors (Levinson, 2005; Shadrina et al., 2018; Fan et al., 2021; Radjabzadeh et al., 2022).

An emerging area of research is focused on evaluating the effect of microbial communities within the human body on mental health (Radjabzadeh et al., 2022; Xiong et al., 2023). Among body systems, the gut microbiome has been extensively studied for its interactions with various physiological processes (Yano et al., 2015; Liu et al., 2022; Radjabzadeh et al., 2022). The gut interacts with the brain via the gut-brain axis by influencing serotonin production as well as influencing inflammation and immune responses within the body, which have been increasingly recognized as factors in psychiatric disorders such as depression and schizophrenia (Dinan and Cryan, 2017; Sharon et al., 2019; Brown and Liu, 2021). Imbalances in the gut microbiome, known as dysbiosis, have also been implicated in various psychiatric conditions (Dinan and Cryan, 2017; Sharon et al., 2019; Brown and Liu, 2021; Lazarini et al., 2022). Research indicates that individuals with depression and anxiety often have reduced microbial diversity, which can contribute to increased inflammation and disrupted neurotransmitter production (Belkaid and Hand, 2014; Dinan and Cryan, 2017; Liu et al., 2022; Xiong et al., 2023). Similarly, altered gut microbiota have been linked to schizophrenia, possibly due to their role in neuroinflammation and dopamine signaling (Belkaid and Hand, 2014; Dinan and Cryan, 2017; Lazarini et al., 2022; Xiong et al., 2023).

The nasal microbiome is emerging as another important factor in psychiatric health (Lazarini et al., 2022; Mousa et al., 2022). The olfactory bulb, which is anatomically connected to the nasal cavity, may provide a pathway through which microbial influences contribute to neuroinflammation, immune responses, and neurotransmitter regulation (Lazarini et al., 2022; Mousa et al., 2022; Xie et al., 2022). Similarly, it is hypothesized that microbial communities are not isolated from one another within the body (Mousa et al., 2022). This would imply that microbial changes or dysbiosis in one community can impact other communities. Therefore, the nasal microbiome may also impact mental health if not directly, then by impacting changes within the gut microbiome, thereby influencing psychiatric symptoms indirectly (Lazarini et al., 2022; Mousa et al., 2022; Xie et al., 2022). Current literature addressing the association between nasal microbiota and psychiatric health remains inconclusive. However, existing evidence suggests that the nasal microbiome may be involved in neurodegenerative diseases, such as Parkinson’s disease (Xie et al., 2022; Hu et al., 2024). However, without a clearer understanding of these molecular mechanisms, the development of more precise interventions, whether pharmacological, microbial, or lifestyle-based, remains limited.

To address this gap, we aimed to evaluate whether there were microbial differences, both in diversity and abundance, between firefighters with and without psychiatric symptomology for PTSD, anxiety, and/or depression. This population not only faces elevated risks of psychiatric conditions but also offers a relatively homogeneous group in terms of exposure to specific stressors and occupational hazards (Wagner et al., 2021; Russ, 2022). In addition, we aimed to identify microbial changes before compared to after exposure to a fire suppression event.

### 1.1 Study hypotheses

We hypothesized that specific microbial abundances of the nasal microbial community at different levels of classification (i.e., phylum, class, order, etc.) would be associated with psychiatric outcomes. We also hypothesized that specific taxa would be detected at different abundances before and after exposure to a fire suppression event.

## 2 Methods

### 2.1 Study design and sample

We conducted a prospective observational study that included both cross-sectional analyses of associations between the nasal microbiome and psychiatric outcomes (anxiety, depression, and PTSD) and repeated measures pre-post analyses evaluating microbial changes before and after a fire suppression event. Firefighters were recruited from December 2023-May 2024 from 14 fire stations within the Houston Fire Department. Firefighters were sampled based on availability during a random emergency fire incident. Inclusion criteria included active-duty firefighter status, aged 18 or older, completion of the standardized mental health questionnaire, and availability for both sampling time points (before and after the fire suppression). The fire chief informed the field team when a fire suppression occurred, as fire departments receive calls randomly. The team typically arrived at the fire scene within 30–45 minutes. The study was approved by the University of Texas Health Science Center at Houston Committee for the Protection of Human Participants (HSC-SPH-23-0929) and the University of Oklahoma Health Campus Institutional Review Board (IRB:18039). All participants signed the informed consent form prior to initiating study activities.

### 2.2 Nasal microbiome sample collection and sequencing

Nasal microbiome samples were collected using sterile nasal swabs (R1107 Zymo Research, Irvine, CA). Samples were collected from the anterior nares (nostrils) following a standardized protocol to minimize variability. According to the protocol, the study participant would rotate the swab tip clockwise 15 times at the entrance of each nostril, ensuring contact with the nasal membrane. The duration of the collection was approximately 90 seconds in total. Each swab was immediately stored at room temperature in DNA stabilizing buffer and sent to the laboratory for processing. DNA extraction from the nasal swabs was performed using Qiagen DNeasy PowerSoil Pro Kit following manufacturer’s instructions. The extracted microbial DNA was analyzed for quality using Nanodrop and was stored at -80 °C until further analysis. Total microbial DNA from each sample was amplified using PCR. Briefly, the 16S V1V3 rDNA region was amplified by PCR and sequenced on the Illumina MiSeq platform using the 2 x 300bp protocol. Primers used for amplification contain adapters for MiSeq sequencing and single-index barcodes incorporated into the reverse primer so that resulting PCR products may be pooled and sequenced directly, targeting an average per/sample yield of 25,000 read pairs. Primers used for the amplification of the V1V3 region were 27F: AGAGTTTGATYMTGGCTCAG and 534R: ATTACCGCGGCKGCTGG. All samples were sequenced at Baylor College of Medicine Alkek Center for Metagenomics and Microbiome Research.

### 2.3 Bioinformatics processing

Raw data files in binary base call (BCL) format were converted into FASTQs and demultiplexed based on the single-index barcodes using the Illumina ‘bcl2fastq’ software. Demultiplexed read pairs underwent an initial quality filtering using bbduk.sh (BBMap, version 38.82), removing Illumina adapters, PhiX reads and reads with a Phred quality score <15 and length <100 bp after trimming. Quality controlled reads were then merged using bbmerge.sh with merge parameters: maxstrict=t, qtrim=t, trimq=15. Merged reads were further filtered via vsearch using the following parameters: max expected error rate = 0.001, min length = 450bp and max length 550bp (Rognes et al., 2016). All reads were then combined into a single FASTA file for further processing using UPARSE (Edgar, 2013). Briefly, resulting reads were clustered into Operational Taxonomic Units (OTUs) at similarity cutoff 97% using an in-house stepwise approach that includes chimera filtering. Reads were first dereplicated using the vsearch ‘derep_fulllength’ option, recording the size to identify singletons (Rognes et al., 2016). Dereplicated non-singleton reads were clustered through an iterative stepwise manner in increments of 0.4% using the usearch70 ‘cluster_otus’ function (Edgar, 2010). Singletons were mapped back to the dereplicated reads using usearch70 ‘-usearch_global’ at a 99% identity and mapped reads were added to an output file (Edgar, 2010). The final output was run through usearch70 ‘uchime_ref’ against the GOLD database v5, using only the positive-sense strand and allowing for no chimeras (Kyrpides, 1999; Edgar et al., 2011). The OTU centroids were then mapped against an optimized version of the latest current SILVA Database containing only sequences from the appropriate variable region of the 16S rRNA gene to determine taxonomies using the usearch70 ‘usearch_global’ function, specifying the identity threshold to 97% (Edgar, 2010; Quast et al., 2013). Abundances were recovered by mapping demultiplexed reads to the OTU file, creating an OTU table in biom format and removing chimeric reads. Phylogeny information contained in the biom file was generated by aligning the centroid sequences with MAFFT and creating a tree via FastTree (Price et al., 2010; Nakamura et al., 2018). The biom file was summarized, recording the number of reads per sample, and merged with a file that is generated for the overall read statistics, to produce a final summary file with read statistics and taxonomy information. Sample profiles were then classified with their taxonomic make-up reported. Alpha diversity (within-sample microbial diversity) was quantified using Observed Operational Taxonomic Units (OTUs). Differentially abundant taxa were identified to ascertain the most common and prevalent taxa across the samples. These comparisons were done with the samples at the Phylum, Class, Order, Family, and Genus levels of classification.

### 2.4 Mental health outcome assessment

Upon enrollment, participants completed a detailed, standardized questionnaire that included validated psychiatric assessment tools as well as questions on demographic and occupation-specific factors of age in years (continuous), sex (male, female), race (Asian, African American, Native American, White), body mass index (measured continuously (kg/m (Martin et al., 2017)) and classified into normal (<18.5-24.9), overweight (25-29.9), and obese (≥ 30)), and fire service characteristics such as total years of service (continuous), and department rank (Captain, Firefighter, Instructor). The mental health assessment tools included 1) Center for Epidemiological Studies-Depression scale (CES-D) (Radloff, 1977), 2) PTSD checklist from the DSM-5 (PCL-5) (Bliese et al., 2008), and 3) Anxiety Sensitivity Index (ASI-3) (Taylor et al., 2007). Each of these tools use Likert scale questions regarding recent symptoms (during the past week/month) to produce an overall score for each of the psychiatric outcomes of interest. Participants were classified into binary outcomes (present or absent) for each psychiatric condition based on established cutoff scores for each psychiatric tool: 1) Depression: CES-D score ≥ 16 (Radloff, 1977), 2) PTSD: PCL-5 score ≥ 31 (Bliese et al., 2008), and 3) Anxiety: ASI-3 score ≥ 17 (Taylor et al., 2007).

### 2.5 Statistical analysis

We summarized demographic and psychiatric information of the participants using frequencies and proportions for categorical variables and means and standard deviations for continuous variables. Samples taken pre-fire event were used for all mental health analyses. Differences in alpha diversity (within-sample diversity) between groups with and without psychiatric classifications were assessed by using t-tests or Mann-Whitney U tests, depending on data distribution (i.e., normal or non-normal distribution). Beta diversity (between-sample microbial diversity) was assessed using Bray-Curtis distances calculated from rarefied OTU counts. Principal Coordinate Analysis (PCoA) was used to visualize sample clustering by psychiatric group, and statistical differences in community composition were tested using a non-parametric statistical test, Permutational Multivariate Analysis of Variance (PERMANOVA). Heatmaps were generated as descriptive visual aids to illustrate microbial abundance patterns.

Differential abundance analysis was conducted using univariate logistic regression models, testing each psychiatric binary outcome against microbial abundance at each taxonomic level. Regression results were corrected for multiple comparisons using the Benjamini-Hochberg (B-H) procedure to control the false discovery rate (FDR). A directed acyclic graph (DAG) was constructed to clarify and evaluate potential confounding pathways between microbial composition and psychiatric outcomes. The resulting minimally sufficient set for adequate adjustment included age and years of service in the occupation (**Supplementary Figure 1**). Taxa that remained significant after FDR correction were then included in multivariable logistic regression models, adjusting for age and years of service.

Because psychiatric outcomes can vary in severity across individuals, we also examined linear severity scores for each outcome to determine whether symptom intensity was associated with specific taxa. For continuous psychiatric scores, we used univariate linear regression to test the association between microbial abundance and symptom severity. We evaluated the assumptions of linearity, homoscedasticity, and normality for all linear regression models using residual diagnostics, Q-Q plots, and the Shapiro-Wilk normality test. Both PTSD and anxiety scores were square root transformed to meet linear regression assumption requirements.

To evaluate changes in microbial composition in response to fire suppression, we used linear mixed models with a random effect for the participant to account for repeated measures (pre- and post-exposure). These models estimated within-subject changes in taxon abundance at each classification level. Taxa were tested with B-H correction. Significant taxa after B-H correction were then reported with covariate-adjusted estimates and 95% confidence intervals. For linear mixed models, residual diagnostics were evaluated to assess normality, homoscedasticity, and model fit assumptions. Alpha and beta diversity were also determined between pre and post samples in the same manner described above.

All regression analyses report estimates as beta coefficients (and subsequent 95% CIs) and a p-value < 0.05 was used as the threshold for statistical significance in all models, with appropriate multiple testing correction based on B-H adjusted p-values < 0.05. Analyses were conducted using R version 4.4.0 (R Core Team, Vienna, Austria).

## 3 Results

A total of 34 participants were included in the analysis and completed all study activities. The average age of the participants was 41 years (SD: 8) and the majority were male and White (91% and 82%, respectively). Approximately one quarter of the participants (26%) were at the rank of captain and the average number of years of service was 15 years among the firefighters (**Table 1**). Of the participants included, 47% met the classification score for depression according to the CES-D criteria (Radloff, 1977). Fewer participants met the classification criteria for PTSD and anxiety according to the PCL-5 and ASI-3 (12% and 18%, respectively) (Taylor et al., 2007; Bliese et al., 2008).

**Table 1:** Demographic and psychiatric characteristics for Texas firefighters.

Categorical variables (N = 34)	N (%)
Sex	
Male	31 (91.2%)
Female	3 (8.8%)
Race	
Asian	2 (5.9%)
Black/African American	3 (8.8%)
Native American	1 (2.9%)
White	28 (82.4%)
BMI Classification	
Normal	2 (5.9%)
Obese	20 (58.8%)
Overweight	12 (35.3%)
Rank	
Captain	9 (26.5%)
Firefighter	22 (64.7%)
Instructor	3 (8.8%)
Depression	
Depressed	16 (47%)
Not Depressed	18 (53%)
Anxiety	
Anxiety	6 (18%)
No Anxiety	28 (82%)
PTSD	
PTSD	4 (12%)
No PTSD	30 (88%)
Continuous Scores	Mean (SD)
Age (years)	41 (8)
BMI	29.9 (3.6)
Years of Service	15 (8)
Depression Score	16.3 (6.1)
Anxiety Index Score	9 (8)
PTSD Score	13 (14)

BMI, body mass index; PTSD, post-traumatic stress disorder; SD, Standard deviation.

### 3.1 Alpha and beta diversity

**Figure 1:**
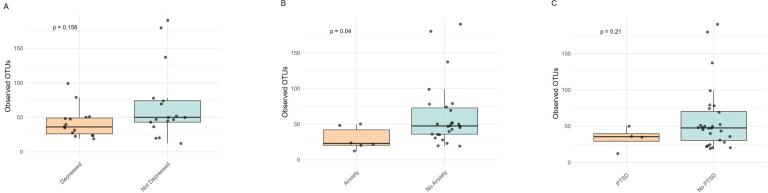
**(A)** Comparison in Alpha diversity between participants with and without Depression based on Operational Taxonomic Units (OTUS). **(B)** Comparison in Alpha diversity between participants with and without Anxiety based on Operational Taxonomic Units (OTUs). **(C)** Comparison in Alpha diversity between participants with and without PTSD based on Operational Taxonomic Units (OTUS).

**Figure 2:**
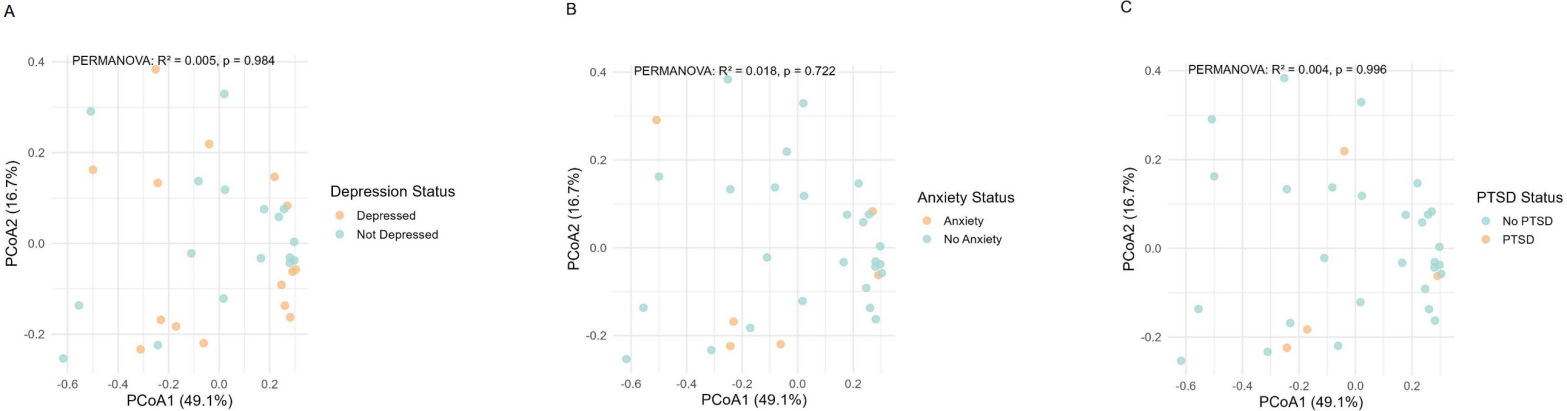
**(A)** Comparison in Beta diversity between participants with and without Depression. **(B)** Comparison in Beta diversity between participants with and without Anxiety. **(C)** Comparison in Beta diversity between participants with and without PTSD.

As shown in **Figure 1A**, participants with psychiatric outcomes generally demonstrated lower alpha diversity, as measured by OTUs, compared to participants without psychiatric classifications. The largest difference was observed for anxiety status, where participants with anxiety had significantly lower alpha diversity relative to participants without anxiety (p = 0.04, **Figure 1**). Although participants with depression and PTSD also showed lower overall diversity, these differences did not reach statistical significance. Bray-Curtis PCoA plots for depression, anxiety, and PTSD outcomes are shown in **Figure 2A**. PERMANOVA analyses did not identify statistically significant differences in beta diversity between participants with and without psychiatric classifications.

### 3.2 Microbial differences between outcomes and symptom severity

In our analysis of microbial composition across psychiatric classifications comparing relative abundances by taxonomic level, we visually observed differences in abundance for taxa including *Firmicutes* and *Actinobacteriota* (phylum level) and *Staphylococcaceae* and *Carnobacteriaceae* (family level) between participants with and without our psychiatric outcomes of interest (**Supplementary Figures 2–4**). These visual patterns were subsequently evaluated using regression-based statistical analyses. In our logistic regression analyses performed at each taxonomic level from phylum to genus, we observed significant differences in relative abundance by psychiatric condition for multiple families and genera. The genus *Hydrotalea* was significantly associated with PTSD status in the covariate-adjusted model (*β* = 0.59, 95% CI: 0.06 to 1.27, p = 0.042) (**Table 2**). Higher relative abundance of *Ruminococcus* was associated with a significantly increased odds of anxiety (*β* = 0.70, 95% CI: 0.18 to 1.48, p = 0.027), indicating that participants with greater abundance of *Ruminococcus* were more likely to be classified as having anxiety after adjusting for age and years of service. When considering analyses across taxonomic levels, results varied. Both the *Ruminococcus* genus (*β* = 0.68, p = 0.023) and the *Ruminococcaceae* family (*β* = 0.64, p = 0.026) were significantly associated with anxiety status (**Supplementary Tables 4.3**, **5.3**). However, no other genera from the *Ruminococcaceae* family were significant. Also of note, the *Ruminococcaceae* family is within *Clostridiales* order, which was not significant, while the order *Oscillospirales* was (*β* = 0.62, p = 0.028) (**Supplementary Table 3.3**). The logistic regression results did not reveal any significant microbes based on depression at any level.

**Table 2:** Unadjusted and adjusted logistic and linear regression results reaching significance at the genus taxonomic level among three psychiatric outcomes.

Outcome	Genus	Unadjusted estimate (95% CI)	B-H p-value	Adjusted estimate (95% CI)	p-value
Logistic regression					
PTSD	*Hydrotalea*	0.47 (0.01-0.99)	0.043	0.59 (0.06-1.27)	0.042
Anxiety	*Ruminococcus*	0.68 (0.19-1.43)	0.023	0.70 (0.18-1.48)	0.027
Linear regression					
Depression	*Chyrseomicrobium*	4.55 (1.71-7.40)	0.003	4.69 (1.75-7.62)	0.003
Depression	*Exiguobacterium*	3.90 (1.08-6.72)	0.008	4.07 (1.13-7.01)	0.009
Depression	*Cupriavidus*	3.25 (0.93-5.57)	0.008	3.42 (1.02-5.83)	0.007
Depression	*Ruminococcus*	1.89 (0.85-2.94)	0.001	2.05 (0.97-3.13)	0.001
Depression	*Dermabacter*	1.49 (0.41-2.57)	0.008	1.50 (0.37-2.63)	0.011
Depression	*Aerococcus*	1.18 (0.41-1.95)	0.004	1.22 (0.43-2.02)	0.004
PTSD*	*Citrobacter*	0.22 (0.02-0.42)	0.029	0.26 (0.06-0.47)	0.013
Anxiety*	*CL500–29 marine*	0.14 (0.004-0.28)	0.044	0.17 (0.03-0.31)	0.021
Anxiety*	*Parabacteroides*	0.04 (0.002-0.08)	0.039	0.05 (0.01-0.09)	0.011

In analyses of continuous outcomes, we observed significant differences in relative abundance of microbes at the genus level comparing depression, PTSD, and anxiety scores on a continuous scale (**Table 2**). For each unit increase in relative abundance of *Aerococcus*, the depression score increased by 1.22 points (95% CI: 0.43 to 2.02, p-value = 0.004) after adjusting for age and years of service, while the depression score increased by 4.69 points (95% CI: 1.75 to 7.62, p = 0.003) with each unit increase in relative abundance of *Chyrseomicrobium* after covariate adjustment. Similarly, there were genera significantly associated with PTSD and anxiety scores. Individuals with greater *Citrobacter* abundance had significantly higher PTSD symptoms severity after covariate adjustment (*β* = 0.26, 95% CI: 0.06-0.47, p-value = 0.013) while individuals with greater *Parabacteroides* was associated with significantly increased anxiety score severity after covariate adjustment (*β* = 0.05, 95% CI: 0.01-0.09, p-value = 0.011), with both scores transformed by taking their square root to meet linear regression assumption requirements.

**Figure 3:**
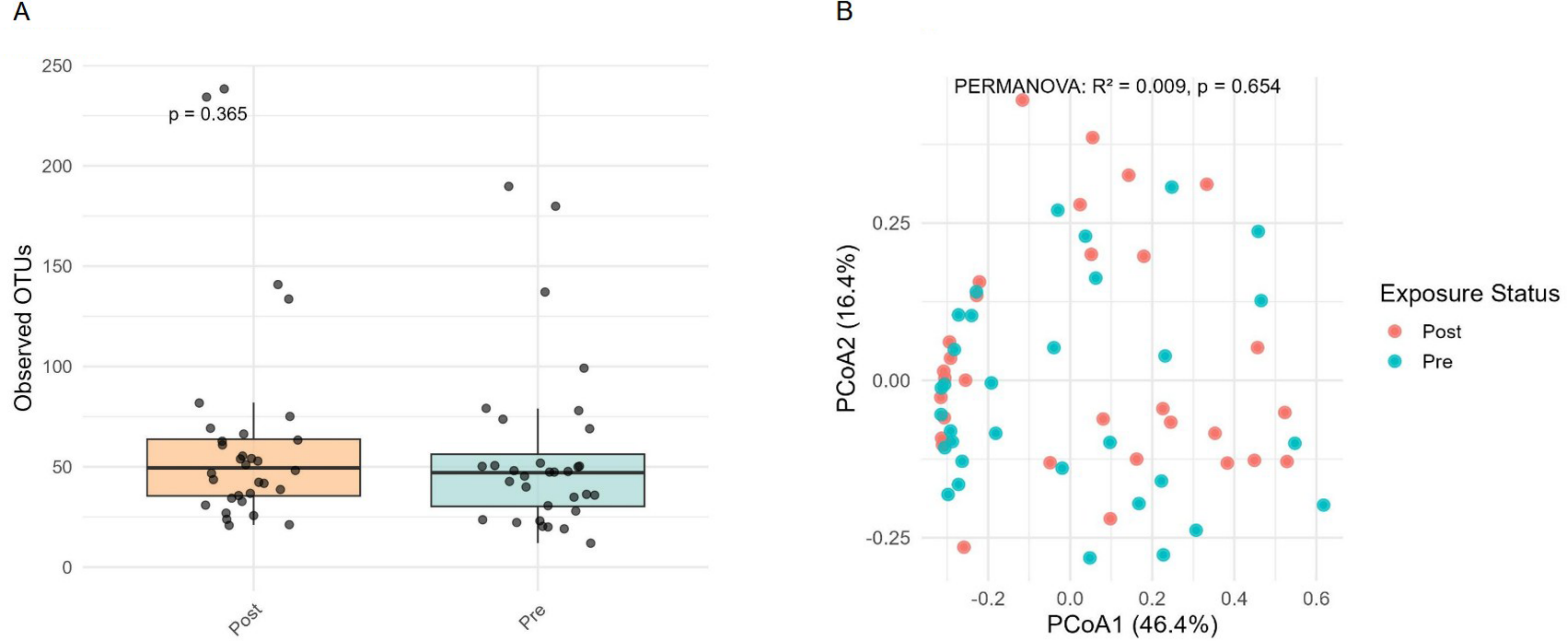
**(A)** Alpha diversity differences between pre and post fire call samples. **(B)** Beta diversity differences between pre and post fire call samples PCOA - Bray-Curtis (Exposure Status).

No significant differences in overall alpha or beta diversity were observed between pre- and post-fire suppression samples (**Figure 3A**), suggesting that acute fire suppression exposure did not substantially alter overall microbial diversity measures. However, taxon-specific analyses identified several genera and higher taxonomic classifications that differed significantly following suppression exposure. For instance, when evaluating linear mixed models, the genus *Enhydrobacter* increased in abundance after the fire suppression by 2.08 units (95% CI: 0.80, 3.46, p-value = 0.004), while the genus *Hymenobacter* decreased by 1.25 units (95% CI: -2.22, - 0.27, p-value = 0.017) in covariate-adjusted models (**Table 3**). Similarly, the family *Hymenobacteraceae* (*β* = -1.28, B-H p-value = 0.013), the class *Verrucomicrobiae* (*β* = -0.28, B-H p-value = 0.030), and the phylum *Verrucomicrobiota* (*β* = -0.28, B-H p-value = 0.030) were all significantly lower following the fire suppression event (**Supplementary Tables 1.7, 2.7, 4.7**, respectively). Relative abundance summaries across taxonomic levels, including phylum-level pre- and post-fire suppression comparisons, are presented in **Supplementary Tables 1**–**5**. Relative abundance between both time points was also illustrated via heatmap (**Supplementary Figure 5**).

**Table 3:** Unadjusted and adjusted linear mixed model regression results reaching significance at the genus taxonomic level comparing microbial differences pre and post fire suppression.

Genus	Univariate estimate (95% CI)	B-H p-value	Adjusted estimate (95% CI)	p-value
*Enhydrobacter*	2.09 (0.80 – 3.45)	0.008	2.08 (0.80 – 3.46)	0.004
*Hymenobacter*	-1.25 (-2.23 – -0.27)	0.015	-1.25 (-2.22 – -0.27)	0.017
*Unc. OTU0098*	-13.13 (-24.96 – -1.25)	0.035	-13.14 (-24.97 – -1.25)	0.035

## 4 Discussion

This study represents a novel investigation into the potential relationship between the nasal microbiome and psychiatric symptoms within firefighters. Our findings suggest that specific microbial taxa are associated with symptoms of depression, anxiety and PTSD, with notable differences in microbial diversity and composition between participants with and without those psychiatric outcomes. These findings provide preliminary evidence supporting the hypothesis that microbial communities may play a role in mental health outcomes, especially within populations that work in high-stress occupations (Foster and McVey Neufeld, 2013; Kelly et al., 2016; Rea et al., 2016). We found that alpha diversity was significantly lower in individuals with anxiety when compared to participants without anxiety, suggesting that reduced microbial diversity in the nasal cavity may be linked to psychological distress/symptoms. This coincides with existing research on the gut microbiome, where lower overall microbial diversity has been associated with negative mental health outcomes (Kelly et al., 2016). While alpha diversity was also lower in participants with depression and PTSD, these differences were not statistically significant.

Among specific taxa, the genus *Ruminococcus* was significantly more abundant in individuals with anxiety. This finding is consistent with previous research in humans reporting elevated levels of *Ruminococcus* in stress-related psychiatric conditions, including anxiety and depression (Jiang et al., 2018; Kumar et al., 2023). *Ruminococcus* has been previously studied in the context of gut health and has been associated with inflammatory properties, which supports a biologically plausible role in the microbiome-brain interactions related to mental health (Kumar et al., 2023). However, other research has reported species-specific differences in direction and magnitude of association, highlighting the need for additional research to clarify the role of *Ruminococcus* in mental health outcomes (Cryan and Dinan, 2012; Kumar et al., 2023).

Our analyses using continuous psychiatric scores further supported these findings. For depression scores, increased abundance of several genera, including *Aerococcus*, *Chryseomicrobium*, *Cupriavidus*, *Dermabacter*, *Exiguobacterium*, and *Ruminococcus* were associated with higher depression scores. Furthermore, with regard to PTSD, *Citrobacter* was associated with higher PTSD scores, while *CL500–29* marine group and *Parabacteroides* were associated with higher anxiety scores. These genera may serve as potential biomarkers for depressive, PTSD, and anxiety symptoms, though their underlying biological mechanisms impacting these outcomes remain unclear (Valles-Colomer et al., 2019).

Mounting evidence suggests that microbial dysbiosis can influence inflammation, and in turn, influence mental health outcomes. For example, a firefighter training study found that intense smoke/heat exposures over one week led to shifts in gut microbial composition, as well as an increase in inflammatory biomarkers (Houser et al., 2022). Moreover, dysbiosis accompanied by increased gut inflammation has been linked to mental health symptoms and outcomes such as depression and anxiety (Yoo et al., 2025). In populations with a high frequency of stressful exposures, specific shifts in microbial communities have been associated with greater PTSD symptom severity (Yoo et al., 2025). Notably, an imbalance in nasal bacteria has been linked to various health conditions and is thought to influence the brain through immune signaling and neural pathways (Hashimoto, 2023). Furthermore, the nasal microbiome may impact the broader gut-brain axis by modulating immune responses or producing neurotransmitter-like metabolites that reach the central nervous system (Hashimoto, 2023). Therefore, acute shifts in the nasal microbiota from occupational exposure to fire smoke, like those identified in this study (i.e., the increase in the genus *Enhydrobacter* and the decrease in the genus *Hymenobacter* after the fire suppression call), could plausibly induce inflammatory or stress-related responses that exacerbate psychiatric symptoms, such as PTSD, depression, and/or anxiety.

Despite these novel findings, several limitations need to be acknowledged. First, the cross-sectional design for the analyses evaluating the nasal microbiome and psychiatric symptoms precludes conclusions about temporality, though this was evaluated to a degree with the pre and post fire call analyses. Also, the relatively small sample size used for this study may limit the statistical power and our ability to find other significant taxa. This also limits the generalizability of our findings to other firefighter populations, as our study sample may not be representative of larger firefighter cohorts. The reliance on self-reported psychiatric measures, while validated using established questionnaires, may have introduced social desirability bias due to the stigma often associated with psychiatric outcomes. This, in turn, could have led to some misclassification of participants (likely non-differential) which may have biased results toward the null. Also, as the participants were recruited in a convenience sample, there may be some selection bias if recruits with or without mental health disorders were less likely to participate when asked. Additionally, because this study did not include a non-firefighter health control group, we were unable to determine whether the observed microbial patterns were specific to firefighters or reflective of broader population-level microbial variation. Finally, the sequencing technique used for this study (16S rRNA gene amplicon sequencing), while highly efficient and accurate, did not produce classifications at the lowest taxonomic level of species, with results limited to the genus level. Although more expensive and time consuming, future studies should consider using whole genome sequencing and DNA barcoding methods, which allow for identification at the species level (Ranjan et al., 2016).

## 5 Conclusions

Our findings suggest that differences in nasal microbial composition are potentially associated with psychiatric symptoms among firefighters, particularly anxiety and PTSD-related outcomes. Specifically, we identified several microbial taxa associated with psychiatric symptom severity and observed microbial abundance changes before and after fire suppression exposure. These findings partially support our study hypotheses and suggest that occupational stress and fire-related exposures may influence the nasal microbiome. Future studies with larger sample sizes and healthy comparison groups are needed to further clarify the role of the nasal microbiome in psychiatric health among high-risk occupational groups.

## Data Availability

The raw data supporting the conclusions of this article will be made available by the authors, without undue reservation.
