# Supplementary figures and images for: Nasal cavity microbial makeup and the influence on psychiatric symptoms following fire exposure in firefighters
Source: Front Microbiomes. 2026 Jun 23;5:1832151. doi: 10.3389/frmbi.2026.1832151 (PMC13337945; doi:10.3389/frmbi.2026.1832151)

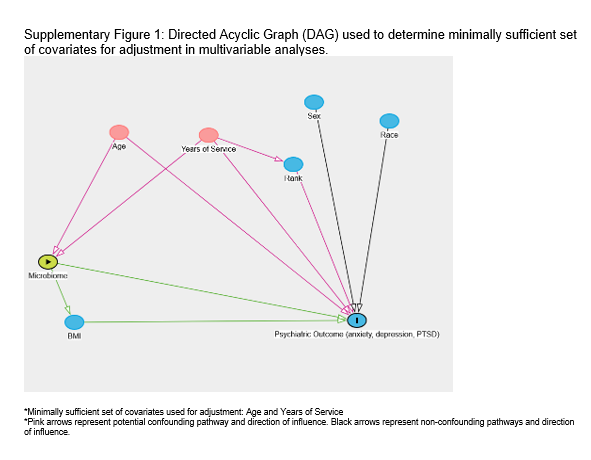

Supplement: Supplementary file 1 [file Image1.png]

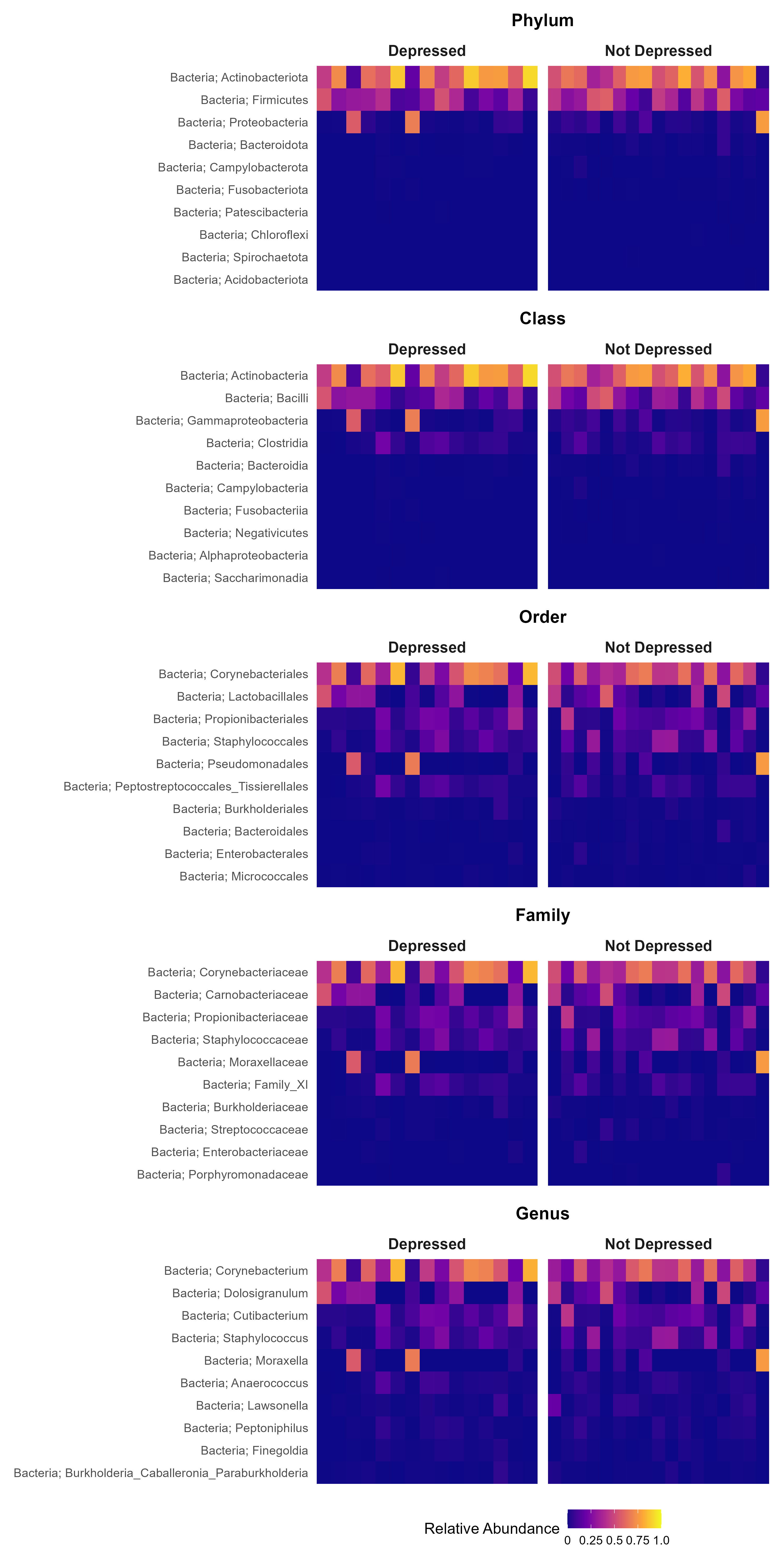

Supplement: Supplementary file 2 [file Image2.jpeg]

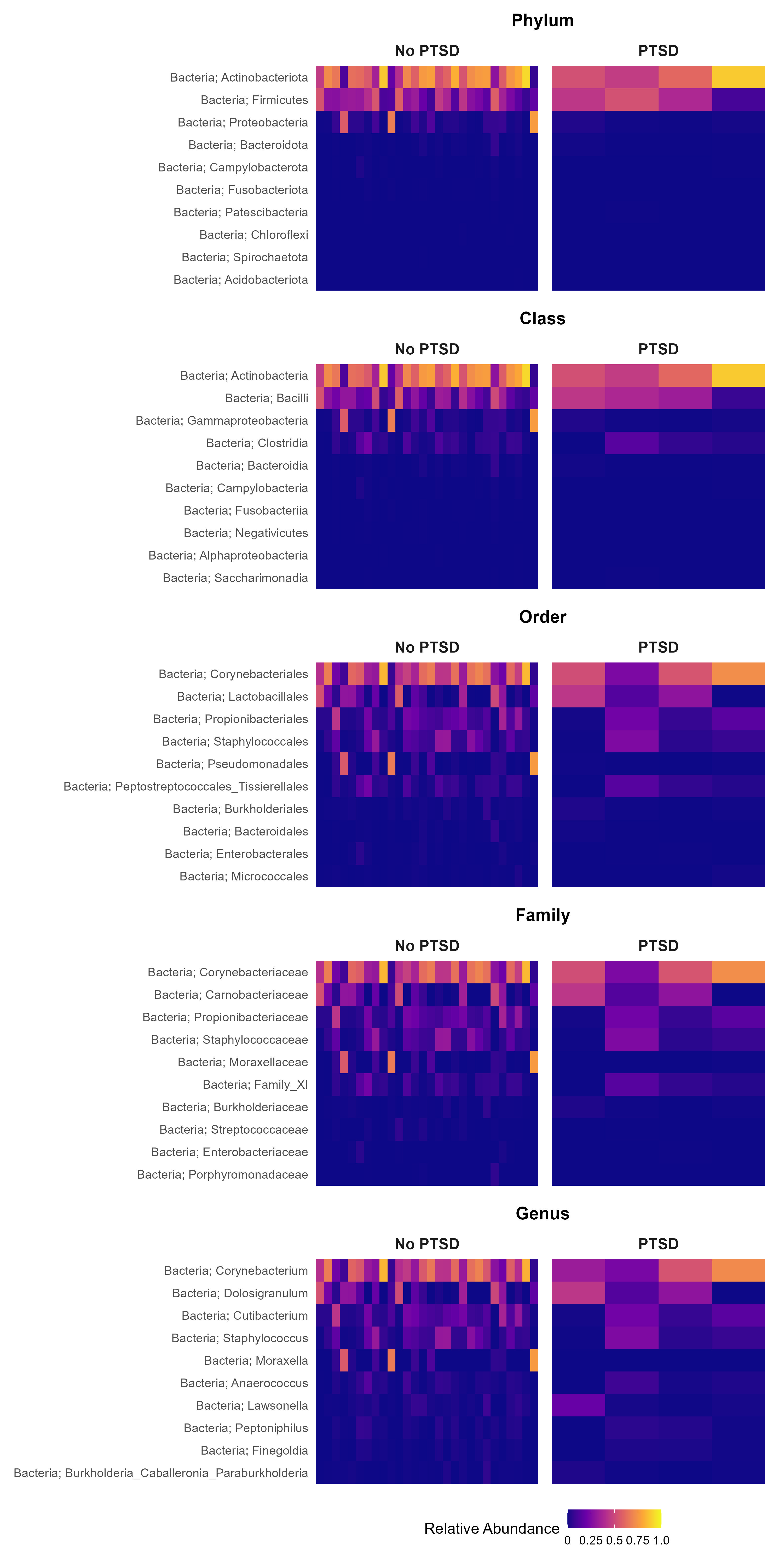

Supplement: Supplementary file 3 [file Image3.jpeg]

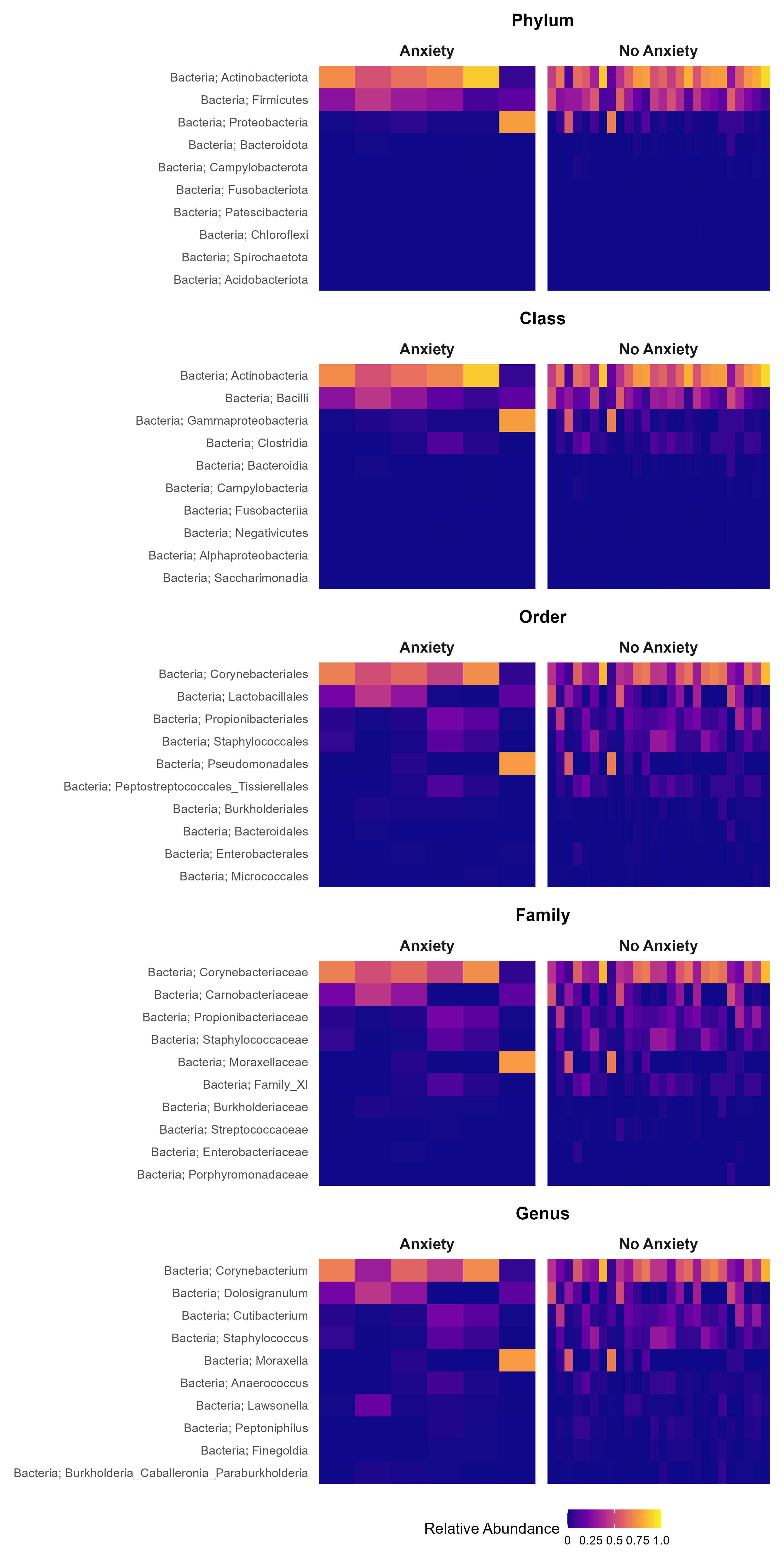

Supplement: Supplementary file 4 [file Image4.jpeg]

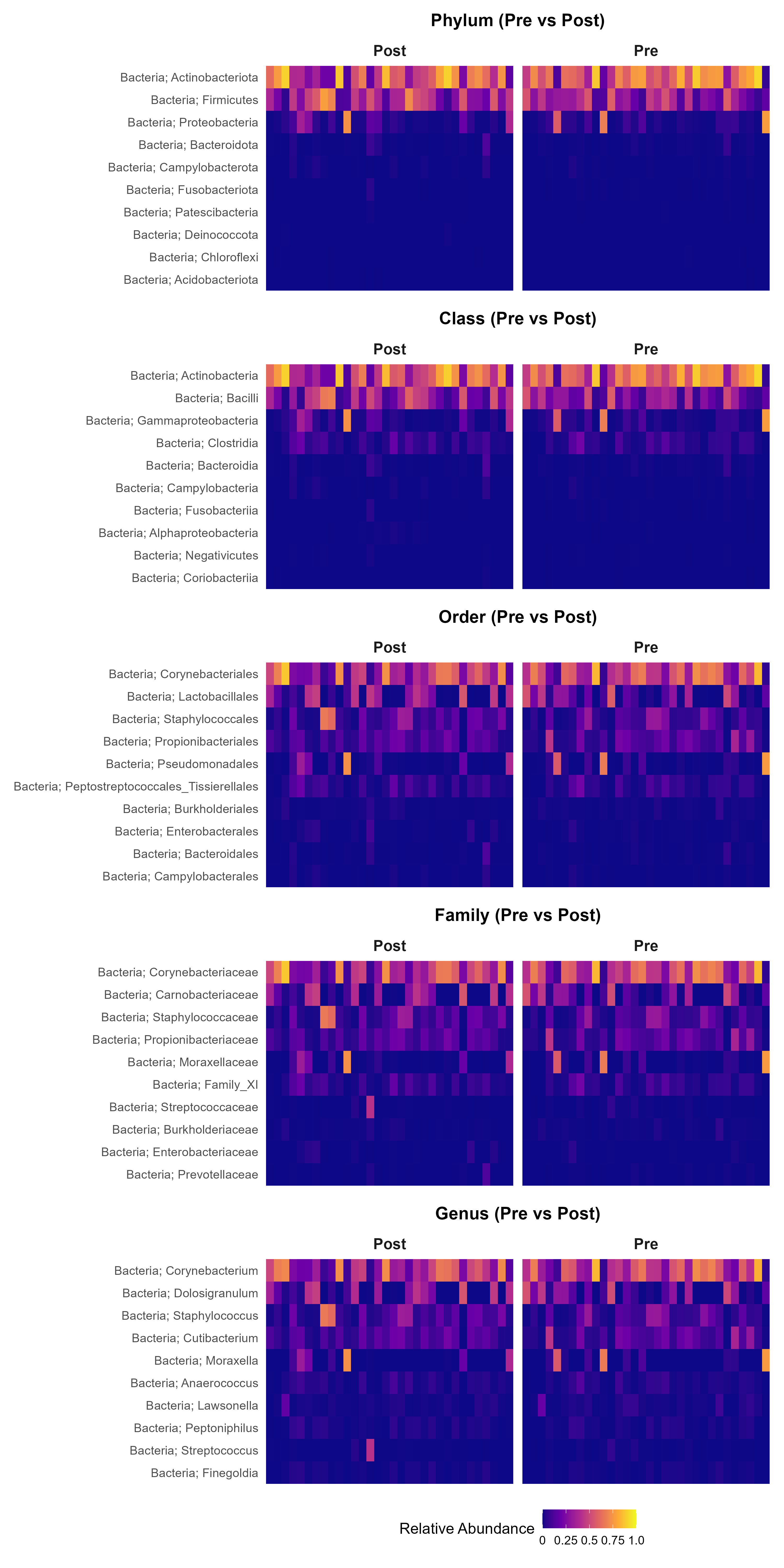

Supplement: Supplementary file 5 [file Image5.jpeg]
